# The intersection of metabolism and inflammation is governed by the intracellular topology of hexokinases and the metabolic fate of glucose

**DOI:** 10.1097/IN9.0000000000000011

**Published:** 2022-10-28

**Authors:** Juan F. Codocedo, Gary E. Landreth

**Affiliations:** 1 Stark Neuroscience Research Institute, Indiana University School of Medicine, Indianapolis, IN, USA; 2 Department of Anatomy, Cell Biology and Physiology, Indiana University School of Medicine, Indianapolis, IN, USA

**Keywords:** hexokinase, glucose

## Abstract

Hexokinases (HKs) catalyze the first and irreversible step of glucose metabolism. Its product, glucose-6-phosphate (G-6P) serves as a precursor for catabolic processes like glycolysis for adenosine 5′-triphosphate (ATP) production and anabolic pathways including the pentose phosphate pathway (PPP) for the generation of intermediaries like nicotinamide adenine dinucleotide phosphate (NADPH) and ribulose-5-P. Thus, the cellular fate of glucose is important not only for growth and maintenance, but also to determine different cellular activities. Studies in immune cells have demonstrated an intimate linkage between metabolic pathways and inflammation, however the precise molecular mechanisms that determine the cellular fate of glucose during inflammation or aging are not completely understood. Here we discuss a study by De Jesus et al that describes the role of HK1 cytosolic localization as a critical regulator of glucose flux by shunting glucose into the PPP at the expense of glycolysis, exacerbating the inflammatory response of macrophages. The authors convincingly demonstrate a novel mechanism that is independent of its mitochondrial functions, but involve the association to a protein complex that inhibits glycolysis at the level of glyceraldehyde 3-phosphate dehydrogenase. We expand the discussion by comparing previous studies related to the HK2 isoform and how cells have evolved to regulate the mitochondrial association of these two isoforms by non-redundant mechanism.

The intimate linkage between metabolic pathways and inflammation has gained substantial attention as it is evident that metabolic enzymes and metabolites do much more than simply provide immune cells with energy in the form of adenosine 5′-triphosphate (ATP). In particular, the family of hexokinases (HKs) that catalyze the first committed step in glucose utilization are a prime example of this immunometabolic crosstalk. After glucose is transported into cells, it is phosphorylated by HKs to produce glucose-6-phosphate (G-6P) which serves as a precursor for glycolysis and also for biosynthetic pathways including the pentose phosphate pathway (PPP) ^[1]^. A central question is how the metabolic fate of glucose is regulated. De Jesus et al ^[2]^ have recently shed substantial new light on this issue, demonstrating that the localization of HK1 within the cell is a critical determinant of the metabolic flux of glucose, governing the generation and distribution of its metabolites and ultimately regulating immune cell functions.

In mammals, five HK isoenzymes have been identified, each with distinct subcellular localization, kinetics, and physiological functions. HK1 is ubiquitously expressed in almost all mammalian tissues and is largely unresponsive to hormones or prevailing metabolic conditions and is considered more of a housekeeping protein. HK2 is detected in adipose, muscles, and myeloid cells whereas HK3 and HK4 (glucokinase) show relatively low expression in mammalian tissues ^[3]^. The fifth member of the family, hexokinase domain-containing protein-1 (HKDC1) is a widely expressed novel HK that plays a role in glucose homeostasis and is involved in the progression of several pathological conditions including cancer ^[4]^. Human HK1 shares with HK2 and HKDC1 a sequence homology close to of 70% ^[4,5]^ and all possess a mitochondrial binding domain (MBD) at their N-terminus that allows the binding to voltage-dependent anion channel (VDAC), an outer mitochondrial membrane protein ^[6,7]^. HK1 is predominantly associated with mitochondria, suggesting that HK1 principally performs a catabolic function, channeling glucose into glycolysis for ATP production ^[3,8]^.

A large body of literature has identified roles for HKs isoforms that extend beyond glucose sensing and phosphorylation. For example, HK2 has been reported to act as a pattern recognition receptor for bacterial particles ^[9]^, to regulate autophagy by direct binding to mammalian target of rapamycin complex 1 (mTORC1) ^[10]^ or by blunting cell death through inhibiting mitochondrial apoptotic pathways ^[11,12]^. Several of these activities are related to their immune function and are dependent of the dynamic nature of HK2 mitochondrial association ^[13]^. The mitochondrial association of HK2 is regulated by different signaling pathways that converge on the activation of AKT serine/threonine kinase 1 (AKT), as only HK2 has a serine-threonine kinase AKT consensus phosphorylation sequence at T473 which regulates its mitochondrial association ^[14]^ (Figure 1, right panel). The mitochondrial interaction of the novel HKDC1 has been reported as essential for its role in liver cancer progression and its dislocation induced mitochondrial dysfunction with the consequential changes in glucose flux ^[16]^. Despite these findings, the molecular mechanisms that govern HKDC1 mitochondrial dynamics under normal and pathological conditions are not yet described.

Recent reports demonstrate that HK1 exhibits pro-survival properties similar to HK2, dictated also by its mitochondrial association ^[17]^, as well as the ability to promote NOD-, LRR- and pyrin domain-containing protein 3 (NLRP3) inflammasome activation. The latter regulates interleukin 1 beta (IL-1β) and IL-18 production in peripheral macrophages in response to lipopolysaccharide (LPS) and ATP stimulation ^[18]^. It was unknown how the intracellular localization of HK1 is governed and how its topology influenced the metabolic and cellular consequences of actions, particularly in immune cells. De Jesus et al ^[2]^ have now described a novel cytosolic gain of function for HK1 that is independent of its enzymatic activity (glucose phosphorylation) but depends on its dislocation of mitochondria and formation of a complex comprised of novel interacting partners, including members of the family of calcium-binding cytosolic S100A8/9 proteins (calprotectin), iNOS, and GAPDH. Importantly, this association results in the nitrosylation of GAPDH, attenuating its activity and redirecting glucose flux to the PPP. Consequently, macrophages display an exaggerated inflammatory response when challenged with LPS. These findings provide new insight into how metabolic fate of glucose is regulated and illuminated the underlying mechanism subserving this effect.

This characterization was done in a novel transgenic mouse, lacking the MBD of the endogenous HK1 (∆E1HK1). As expected, the truncation altered the cellular distribution of HK1 but did not affect its catalytic activity. Importantly, the cytosolic localization of HK1 affects neither the expression, nor the mitochondrial localization of HK2. Interestingly, in bone marrow–derived macrophages (BMDMs) isolated from wildtype mice, HK1 showed a reversible dislocation in response to LPS. Importantly, increased levels of cytosolic HK1 were observed in immune cells of mouse and human conditions associated to inflammation, namely aging and diabetes.

By using ^13^C_6_-glucose tracing metabolomics, the authors revealed that glucose flux was redirected to the PPP after proinflammatory stimulation. This result is not surprising, as similar metabolic consequences have been described for cytosolic HK2 ^[15,19]^ and cytosolic HKDC1 ^[16]^. However, the molecular mechanisms responsible for this “rerouting” of glucose are largely unknown. Interestingly, they observed a reduction in the ^13^C_6_-glucose incorporation of glycolytic intermediates below the level of GAPDH suggesting that the cytosolic localization of HK1 alters glycolysis and increases G-6P incorporation into PPP at the expense of lower glycolysis. This novel function of HK1 induces a “broken glycolysis” which allow the accumulation of metabolites that will drive the increase of the PPP (Figure 1, left panel).

**Figure 1.**
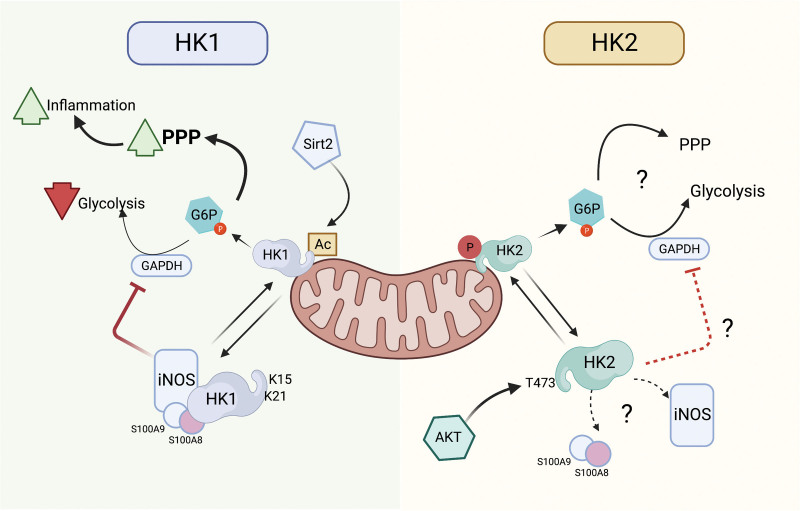
**HK mitochondrial dislocation is regulated by different post-translational mechanisms which regulate the metabolic flux of glucose**. HK1 and 2 contain a mitochondrial binding domain that interact with protein located in the outer membrane of mitochondria. Its product, G-6P, is subsequently metabolized by different pathways including glycolysis and the PPP among others. The balance between these metabolic pathways influences different cellular phenotypes including inflammation. Left panel (modified from De Jesus et al ^[2]^), HK1 mitochondrial association is promoted by acetylation at K15 and 21. The upregulation of deacetylases like Sirt2 induce the dissociation of HK1 which then forms a cytosolic complex with iNOS to inhibit glycolysis at the level of glyceraldehyde 3-phosphate dehydrogenase (GAPDH). Consequently, the metabolic fate of glucose shifts to favor the PPP that in macrophages promote the expression of inflammatory cytokines. Right panel: The association of HK2 is not affected by deacetylases. Instead, early studies have shown that AKT-dependent phosphorylation of T473 promotes its mitochondrial binding. An independent study in cardiomyocytes showed that the overexpression of HK2 promotes the PPP ^[15]^, but the mechanism associated with this shift in the metabolic flux of glucose is unknown. Determination of whether HK2 or other cytosolic HKs can induce the inhibition of GAPDH in different context will be important to understand the level of redundancy of these mechanisms. This figure was created with BioRender.com. AKT: AKT serine/threonine kinase 1; G-6P: glucose-6-phosphate; GAPDH: glyceraldehyde 3-phosphate dehydrogenase; HK: hexokinases; iNOS: inducible nitric oxide synthase; PPP: pentose phosphate pathway.

Because HK1 lacks the AKT consensus site of HK2, the authors explore the molecular mechanism that could direct this phenomenon in macrophages. By using unbiased proteomic analysis, they show that Lys 15 and 21 located in the MBD of HK1 were acetylated and that the acetylation promoted the insertion of the MBD in the hydrophobic mitochondrial outer membrane, resulting in reduction of inflammatory cytokine levels. In the search for the upstream regulator of this process, the authors established that Sirt2, an nicotinamide adenine dinucleotide (NAD)–dependent deacetylase, is likely an important regulator of HK1 mitochondrial association by post-translational modifications of the HK1 MBD.

In summary, De Jesus et al ^[2]^ report a novel mechanism by which cytosolic HK1 directs glucose flux to the PPP by inhibiting glycolysis at the level of GAPDH. This is achieved by its trans-*S*-nitrosylation and consequent GAPDH inhibition (Figure 1, left panel). There is clear evidence that the metabolism of glucose is controlled by the gene expression of different key metabolic enzymes in response to nutrient and oxygen sensing mediated by master regulators of metabolism like hypoxia-inducible factor 1-alpha (HIF-1alpha) and mTOR ^[20]^. The findings of De Jesus et al ^[2]^ identify a new layer of regulation accounting for the control that metabolism has on the inflammatory effects of external stimuli. Perhaps more relevant, the discovery that acetylation of Lys in the MBD of HK1 and deacetylases like Sirt2 can control the mitochondrial dislocation of HK1 reveal a new evolutionary specialization of HKs as theses residues are poorly conserved in HK2. In that way, the mitochondrial localization of these two isoforms is regulated by different post-translational modifications (acetylation and phosphorylation) and upstream signaling enzymes (Sirt2 and AKT, respectively) suggesting that both isoforms can influence the fate of glucose and the immune response by non-redundant mechanisms (Figure 1). Another interesting mechanism that relates HKs localization, metabolic reprograming and immunity was reported in liver carcinogenesis, which is characterized by increased glycolysis and hepatic inflammation ^[21]^. This mechanism is not dependent of post-translational modifications of individual isoforms, but is the consequence of an isoenzyme expression switch between the cytosolic HK4 and mitochondrial HK2 ^[22]^. It is possible to speculate that a similar phenomenon could take place in immune cells that express the mitochondrial isoforms HK1 and HK2 as well as the cytosolic HK3 ^[23]^.

Another source of non-redundancy between isoforms could be associated with non-glucose targets of cytosolic HKs. Considering that cytosolic HK2 and HKDC1 can also redirect G-6P to the PPP ^[15,16]^, it should be important to determine if the mitochondrial dislocation of these enzymes can also influence the inhibition of GAPDH by interacting with cytosolic proteins like calprotectin and iNOS (Figure 1, right panel) in cells where these isoforms are predominant. Similarly, it was recently described that cytosolic HK2 phosphorylates IκBα at T291 inducing its degradation and nuclear factor kappa B (NF-κB) activation-dependent transcriptional upregulation in tumor cells ^[24]^. Determination of whether other cytosolic HKs can regulate the NF-κB pathway by phosphorylation and degradation of its repressor could be important to better understand the level of overlap between their activities in the induction of the immune response. This knowledge could be instrumental in the development of specific metabolic therapies of pathologies associated with dysregulated inflammation.

## Conflicts of interest

The authors declare that they have no conflicts of interest.

## Funding

This work was supported by grant from National Institutes of Health (NIH) (to Gary E. Landreth, RF1AG068400). Juan F. Codocedo was supported by BrightFocus Foundation (A20201166F).

## References

[1] Roberts DJ Miyamoto S. Hexokinase II integrates energy metabolism and cellular protection: Akting on mitochondria and TORCing to autophagy. Cell Death Differ. 2015;22:248–57. doi: 10.1038/cdd.2014.173. 25323588 10.1038/cdd.2014.173 PMC4291497

[2] De Jesus A Keyhani-Nejad F Pusec CM. Hexokinase 1 cellular localization regulates the metabolic fate of glucose. Mol Cell. 2022;82:1261–77.e9. doi: 10.1016/j.molcel.2022.02.028. 35305311 10.1016/j.molcel.2022.02.028 PMC8995391

[3] Wilson JE. Isozymes of mammalian hexokinase: structure, subcellular localization and metabolic function. J Exp Biol. 2003;206:2049–57. doi: 10.1242/jeb.00241. 12756287 10.1242/jeb.00241

[4] Zapater JL Lednovich KR Khan MW. Hexokinase domain-containing protein-1 in metabolic diseases and beyond. Trends Endocrinol Metab. 2022;33:72–84. doi: 10.1016/j.tem.2021.10.006. 34782236 10.1016/j.tem.2021.10.006 PMC8678314

[5] Garcia SN Guedes RC Marques MM. Unlocking the potential of HK2 in cancer metabolism and therapeutics. Curr Med Chem. 2019;26:7285–22. doi: 10.2174/0929867326666181213092652. 30543165 10.2174/0929867326666181213092652

[6] Pusec CM De Jesus A Khan MW. Hepatic HKDC1 expression contributes to liver metabolism. Endocrinology. 2019;160:313–30. doi: 10.1210/en.2018-00887. 30517626 10.1210/en.2018-00887 PMC6334269

[7] Haloi N Wen P-C Cheng Q. Structural basis of complex formation between mitochondrial anion channel VDAC1 and Hexokinase-II. Commun Biol. 2021;4:667. doi: 10.1038/s42003-021-02205-y. 34083717 10.1038/s42003-021-02205-y PMC8175357

[8] John S Weiss JN Ribalet B. Subcellular localization of hexokinases I and II directs the metabolic fate of glucose. PLoS One. 2011;6:e17674. doi: 10.1371/journal.pone.0017674. 21408025 10.1371/journal.pone.0017674 PMC3052386

[9] Wolf AJ Reyes CN Liang W. Hexokinase is an innate immune receptor for the detection of bacterial peptidoglycan. Cell. 2016;166:624–36. doi: 10.1016/j.cell.2016.05.076. 27374331 10.1016/j.cell.2016.05.076 PMC5534359

[10] Roberts DJ Tan-Sah VP Ding EY. Hexokinase-II positively regulates glucose starvation-induced autophagy through TORC1 inhibition. Mol Cell. 2014;53:521–33. doi: 10.1016/j.molcel.2013.12.019. 24462113 10.1016/j.molcel.2013.12.019 PMC3943874

[11] Hinrichsen F Hamm J Westermann M. Microbial regulation of hexokinase 2 links mitochondrial metabolism and cell death in colitis. Cell Metab. 2021;33:2355–66.e8. doi: 10.1016/j.cmet.2021.11.004. 34847376 10.1016/j.cmet.2021.11.004

[12] Pastorino JG Shulga N Hoek JB. Mitochondrial binding of hexokinase II inhibits Bax-induced cytochrome c release and apoptosis. J Biol Chem. 2002;277:7610–8. doi: 10.1074/jbc.M109950200. 11751859 10.1074/jbc.M109950200

[13] Seki SM Gaultier A. Exploring non-metabolic functions of glycolytic enzymes in immunity. Front Immunol. 2017;8:1549. doi: 10.3389/fimmu.2017.01549. 29213268 10.3389/fimmu.2017.01549 PMC5702622

[14] Roberts DJ Tan-Sah VP Smith JM. Akt Phosphorylates HK-II at Thr-473 and increases mitochondrial HK-II association to protect cardiomyocytes. J Biol Chem. 2013;288:23798–806. doi: 10.1074/jbc.M113.482026. 23836898 10.1074/jbc.M113.482026 PMC3745326

[15] McCommis KS Douglas DL Krenz M. Cardiac-specific hexokinase 2 overexpression attenuates hypertrophy by increasing pentose phosphate pathway flux. J Am Heart Assoc. 2013;2:e000355. doi: 10.1161/JAHA.113.000355. 24190878 10.1161/JAHA.113.000355 PMC3886755

[16] Khan MW Terry AR Priyadarshini M. The hexokinase “HKDC1” interaction with the mitochondria is essential for liver cancer progression. Cell Death Dis. 2022;13:660. doi: 10.1038/s41419-022-04999-z. 35902556 10.1038/s41419-022-04999-z PMC9334634

[17] Lauterwasser J Fimm-Todt F Oelgeklaus A. Hexokinases inhibit death receptor-dependent apoptosis on the mitochondria. Proc Natl Acad Sci USA. 2021;118:e2021175118. doi: 10.1073/pnas.2021175118. 34385311 10.1073/pnas.2021175118 PMC8379972

[18] Moon J-S Hisata S Park M-A. mTORC1-Induced HK1-dependent glycolysis regulates NLRP3 inflammasome activation. Cell Rep. 2015;12:102–15. doi: 10.1016/j.celrep.2015.05.046. 26119735 10.1016/j.celrep.2015.05.046 PMC4858438

[19] Sun L Shukair S Naik TJ. Glucose phosphorylation and mitochondrial binding are required for the protective effects of hexokinases I and II. Mol Cell Biol. 2008;28:1007–17. doi: 10.1128/MCB.00224-07. 18039843 10.1128/MCB.00224-07 PMC2223386

[20] Cheng S-C Quintin J Cramer RA. mTOR- and HIF-1α-mediated aerobic glycolysis as metabolic basis for trained immunity. Science. 2014;345:1250684. doi: 10.1126/science.1250684. 25258083 10.1126/science.1250684 PMC4226238

[21] Mansouri A Gattolliat C-H Asselah T. Mitochondrial dysfunction and signaling in chronic liver diseases. Gastroenterology. 2018;155:629–47. doi: 10.1053/j.gastro.2018.06.083. 30012333 10.1053/j.gastro.2018.06.083

[22] Perrin-Cocon L Vidalain P-O Jacquemin C. A hexokinase isoenzyme switch in human liver cancer cells promotes lipogenesis and enhances innate immunity. Commun Biol. 2021;4:217. doi: 10.1038/s42003-021-01749-3. 33594203 10.1038/s42003-021-01749-3 PMC7886870

[23] Seiler K Humbert M Minder P. Hexokinase 3 enhances myeloid cell survival via non-glycolytic functions. Cell Death Dis. 2022;13:448. doi: 10.1038/s41419-022-04891-w. 35538058 10.1038/s41419-022-04891-w PMC9091226

[24] Guo D Tong Y Jiang X. Aerobic glycolysis promotes tumor immune evasion by hexokinase2-mediated phosphorylation of IκBα. Cell Metab. 2022;34:1312–24.e6. doi: 10.1016/j.cmet.2022.08.002. 36007522 10.1016/j.cmet.2022.08.002

